# Detangling Seasonal Relationships of Fecal Contamination Sources and Correlates with Indicators in Michigan Watersheds

**DOI:** 10.1128/spectrum.00415-22

**Published:** 2022-06-22

**Authors:** Amanda M. Wilson, Sherry L. Martin, Marc P. Verhougstraete, Anthony D. Kendall, Amity G. Zimmer-Faust, Joan B. Rose, Melanie L. Bell, David W. Hyndman

**Affiliations:** a Department of Community, Environment & Policy, Mel and Enid Zuckerman College of Public Health, University of Arizona, Tucson, Arizona, USA; b Department of Earth and Environmental Sciences, College of Natural Science, Michigan State University, East Lansing, Michigan, USA; c Southern California Coastal Water Research Project Authority, Costa Mesa, California, USA; d Department of Fisheries and Wildlife, College of Agriculture and Natural Resources, Michigan State University, East Lansing, Michigan, USA; e Department of Epidemiology and Biostatistics, Mel and Enid Zuckerman College of Public Health, University of Arizona, Tucson, Arizona, USA; f Department of Geosciences, School of Natural Sciences and Mathematics, University of Texas, Richardson, Texas, USA; University of Minnesota

**Keywords:** *B. thetaiotaomicron*, *E. coli*, watershed, water quality, fecal indicator, septic systems

## Abstract

Despite the widely acknowledged public health impacts of surface water fecal contamination, there is limited understanding of seasonal effects on (i) fate and transport processes and (ii) the mechanisms by which they contribute to water quality impairment. Quantifying relationships between land use, chemical parameters, and fecal bacterial concentrations in watersheds can help guide the monitoring and control of microbial water quality and explain seasonal differences. The goals of this study were to (i) identify seasonal differences in Escherichia coli and Bacteroides thetaiotaomicron concentrations, (ii) evaluate environmental drivers influencing microbial contamination during baseflow, snowmelt, and summer rain seasons, and (iii) relate seasonal changes in B. thetaiotaomicron to anticipated gastrointestinal infection risks. Water chemistry data collected during three hydroclimatic seasons from 64 Michigan watersheds were analyzed using seasonal linear regression models with candidate variables including crop and land use proportions, prior precipitation, chemical parameters, and variables related to both wastewater treatment and septic usage. Adaptive least absolute shrinkage and selection operator (LASSO) linear regression with bootstrapping was used to select explanatory variables and estimate coefficients. Regardless of season, wastewater treatment plant and septic system usage were consistently selected in all primary models for B. thetaiotaomicron and E. coli. Chemistry and precipitation-related variable selection depended upon season and organism. These results suggest a link between human pollution (e.g., septic systems) and microbial water quality that is dependent on flow regime.

**IMPORTANCE** In this study, a data set of 64 Michigan watersheds was utilized to gain insights into fecal contamination sources, drivers, and chemical correlates across seasons for general E. coli and human-specific fecal indicators. Results reaffirmed a link between human-specific sources (e.g., septic systems) and microbial water quality. While the importance of human sources of fecal contamination and fate and transport variables (e.g., precipitation) remain important across seasons, this study provides evidence that fate and transport mechanisms vary with seasonal hydrologic condition and microorganism source. This study contributes to a body of research that informs prioritization of fecal contamination source control and surveillance strategy development to reduce the public health burden of surface water fecal contamination.

## INTRODUCTION

The financial and public health impacts of fresh water fecal contamination have long been recognized: fecal contamination of freshwater is a public health concern with potential to degrade the quality of irrigation water for food crops (1, 2), groundwater used for drinking water (3), and surface waters that have multiple beneficial uses (4), yielding significant disease and financial burdens. Direct exposure to contaminated surface waters, such as those through recreational activities, can also lead to negative health outcomes (5, 6). For example, an epidemiological study at beaches in Brazil found direct correlations between *Enterococcus* concentrations in water and gastrointestinal illness rates in swimmers (7). Freshwater beach epidemiological studies have correlated Escherichia coli concentrations in water with gastrointestinal illnesses in swimmers (8, 9).

Two commonly utilized methods for investigating fecal contamination sources in surface waters are (i) indicator organisms and (ii) microbial source tracking (MST) of host-specific gene markers. Indicator organisms (e.g., E. coli) are not specific to a particular animal species. MST methods utilize PCR-based methods to quantify specific gene sequences associated with different sources (10–12). One example of MST is the use of single-copy homologues encoding Bacteroides thetaiotaomicron α-1-6 mannanases found in high percentages of human fecal samples and indicative of human fecal contamination when detected in water (13, 14).

Some transport factors influencing fecal contamination in water are well recognized, such as runoff from land into surface waters (15, 16). However, the specific mechanisms of fecal contamination fate and transport and corresponding public health burdens are not well understood. Improving surveillance protocols for prioritizing fecal contamination source control will help to implement evidence-based public health interventions.

Contaminant occurrences and concentrations depend on myriad factors, including contamination sources, hydrologic conditions (17), physical properties of the water, weather (18–20), population density, land use characteristics, and human behavior (21). In temperate regions, some of these parameters are seasonally driven, resulting in water quality differences within any given year. As mentioned previously, precipitation and hydrologic conditions are well-known drivers of fecal contamination. A previous Great Lakes study found inconsistent relationships between fecal indicator bacteria and human-specific markers under low- and high-flow conditions, suggesting that hydrologic conditions play a role in water contamination (20). However, parsing out individual fates and transport mechanisms for specific fecal contaminants can be challenging in complex systems that receive multiple sources of water and pollution.

Relating E. coli and B. thetaiotaomicron fate and transport mechanisms with seasonal environmental changes would improve the understanding of risks posed by fecal contamination in surface waters as different recreational activities vary seasonally. Therefore, this study sought to (i) identify seasonal differences in E. coli and B. thetaiotaomicron concentrations in surface waters, (ii) determine environmental drivers of microbial contamination in baseflow, snowmelt, and summer rain seasons, and (iii) use quantitative microbial risk assessment (QMRA) across seasons to address exposures from recreational activities and associated gastrointestinal illnesses. Quantifying relationships between fecal contamination, land use or environmental parameters, and human health can provide a basis to improve watershed management policies and microbial risk mitigation.

## RESULTS

**FIG 1:**
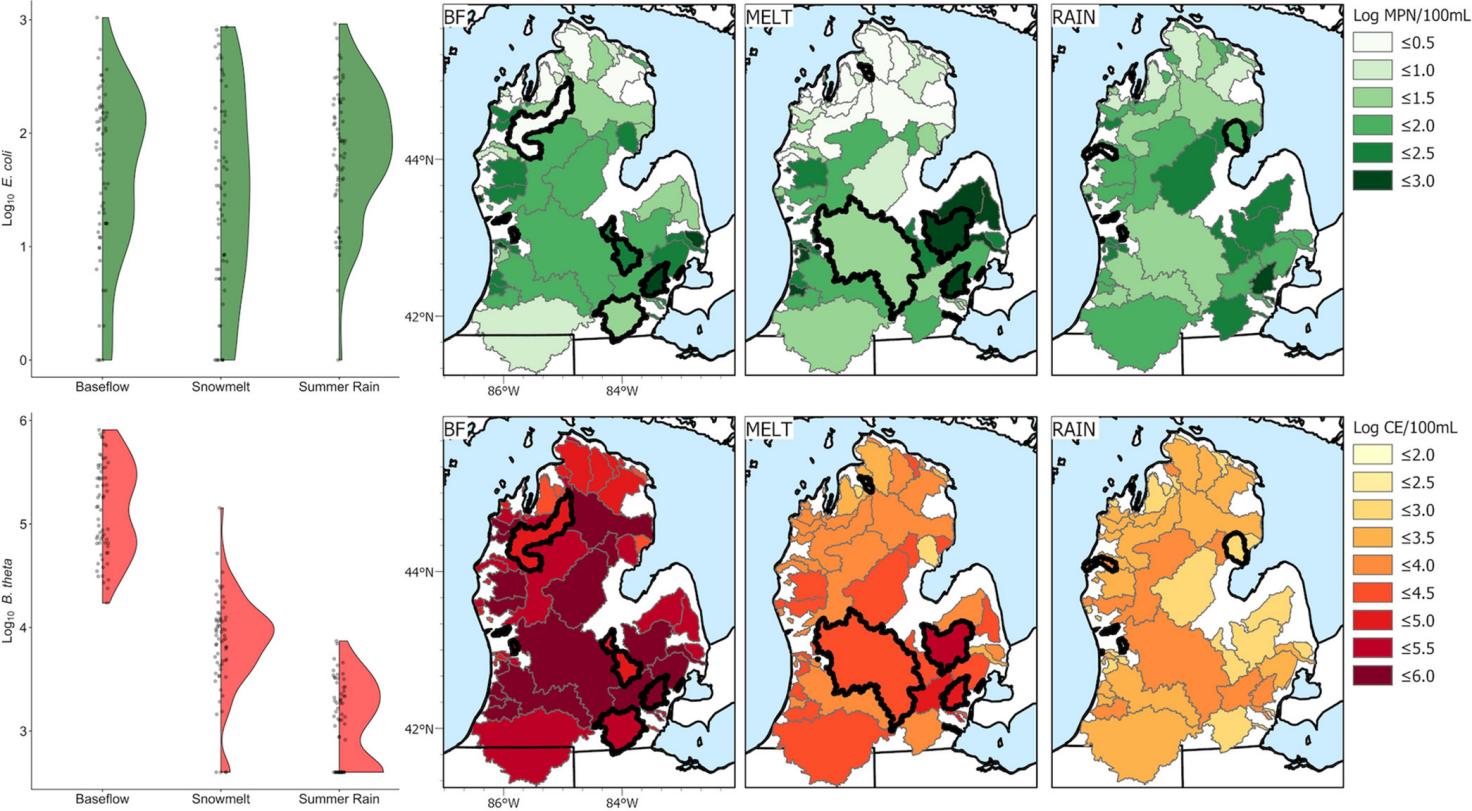
Distributions of B. thetaiotaomicron (*B. theta*) and E. coli log_10_ concentrations across seasons in chronological order of measurement collection (baseflow [BF], snowmelt, and summer rain). Each watershed sampled in this study is shown with corresponding color and gray border. Bolded watersheds were identified as influential watersheds in the sensitivity analysis. Baseflow measurements were collected from 1 to 13 October 2010. Snowmelt and summer rain measurements were collected from 5 to 23 February 2011 and from 1 to 28 June 2011, respectively.

In the 64 watersheds studied across a large (88,000-km^2^) spatial scale, there were significant seasonal differences in log_10_ concentrations of B. thetaiotaomicron (*P* < 0.001), a human fecal source tracking marker (Fig. 1; see also Table S1 in the supplemental material). Mean log_10_ concentrations of B. thetaiotaomicron varied over multiple orders of magnitude. Concentrations were highest during baseflow (5.15 log_10_ cell equivalents [CE]/100 mL), followed by the snowmelt (3.84 log_10_ CE/100 mL) and summer rain (3.07 log_10_ CE/100 mL) seasons (Fig. 1). This trend was routinely measured across watersheds (Fig. 1). In contrast, mean log_10_ E. coli concentrations, while statistically significantly different (*P* < 0.002) across seasons, were within the same order of magnitude (~1 log_10_; baseflow, 1.81 log_10_; snowmelt, 1.53 log_10_; summer rain, 1.93 log_10_), with no strong spatial pattern across seasons (Fig. 1). Most precipitation and chemical parameters were significantly different across seasons (Table S1).

### Notable least absolute shrinkage and selection operator (LASSO)-selected variables and their seasonal trends.

**FIG 2:**
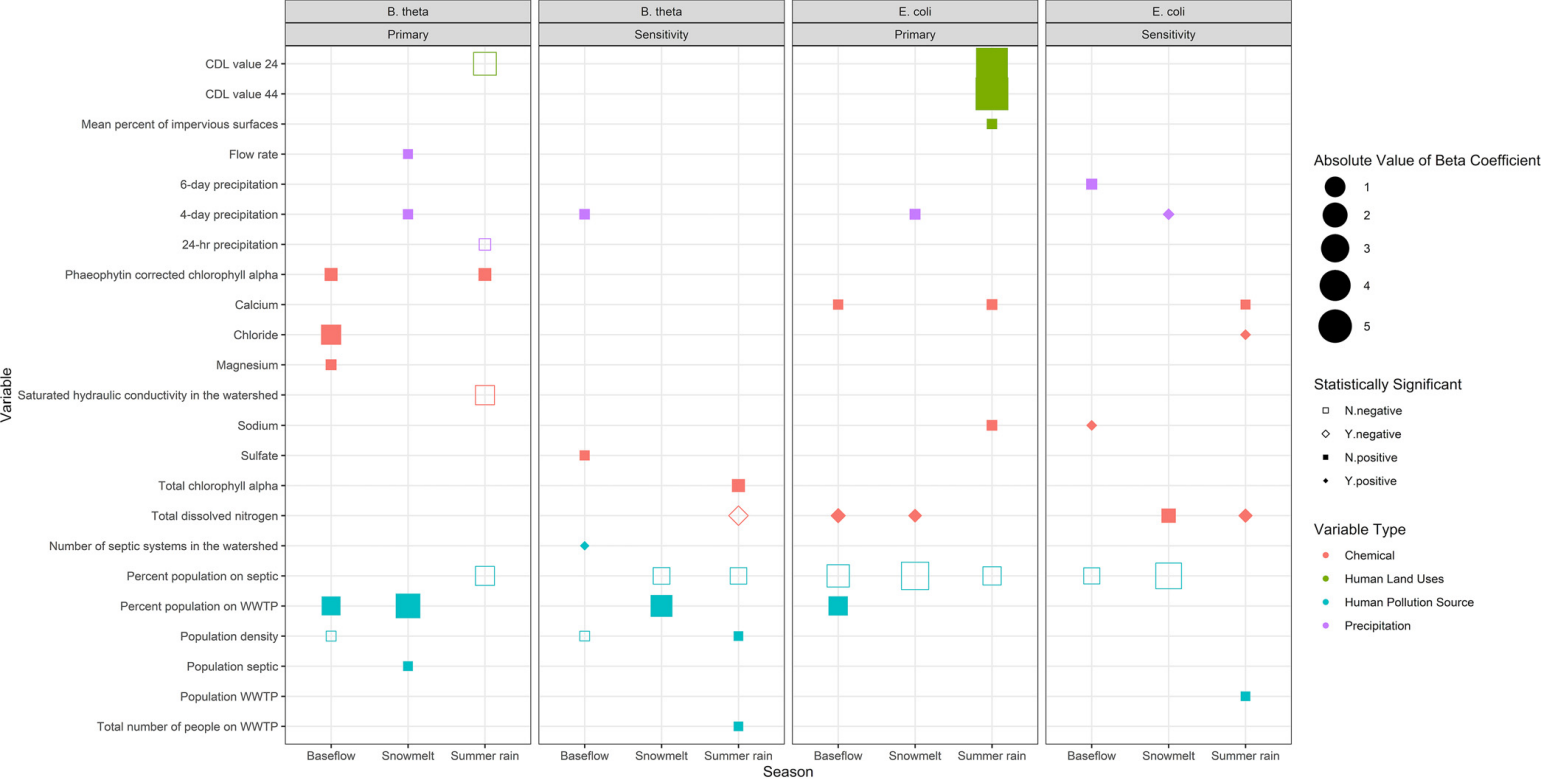
Selected and statistically significant explanatory variables for primary and sensitivity E. coli and B. thetaiotaomicron models. Variable names are described in more detail in Table S2. The total numbers of observations (i.e., watersheds) used in the primary models for E. coli and B. thetaiotaomicron per season were 56, 46, and 60 for the baseflow, snowmelt, and summer rain models, respectively. In sensitivity analysis E. coli models, 2, 3, and 4 influential watersheds were removed from the baseflow, snowmelt, and summer rain data sets, respectively. In the sensitivity analysis B. thetaiotaomicron models, 5, 4, and 2 influential watersheds were removed from baseflow, snowmelt, and summer rain data sets, respectively. Missing data are described in the supplemental material. N.negative, not statistically significant negative relationship; Y.negative, statistically significant negative relationship; N.positive, not statistically significant positive relationship; Y.positive, statistically significant positive relationship.

Total dissolved nitrogen was selected in multiple primary and sensitivity models with statistically significant estimated beta coefficients (Fig. 2). While primarily selected for E. coli, one model also selected total dissolved nitrogen for B. thetaiotaomicron (summer rain, sensitivity). Based on these beta coefficients, the E. coli baseflow model found that an increase of 1 μg/L of total dissolved nitrogen was associated with a 36% $\left[(e^{3.1\times 10^{-1}}\;-\;1)\times 100\text{\%}\right]$ increase in E. coli concentrations (most probable number [MPN]/100 mL). In the snowmelt model, an increase of 1 μg/L in total dissolved nitrogen was related to a 20% $\left[(e^{1.8\times 10^{-1}}\;-\;1)\times 100\text{\%}\right]$ increase in E. coli concentrations (MPN/100 mL) (Table S2).

Other consistently selected variables for all seasons related to B. thetaiotaomicron human-specific fecal contamination sources included WWTP- and septic-related variables (Fig. 2). Two of the human pollution-related variables (e.g., percentage of the population on septic, percentage of the population on WWTPs, number of septic systems in watersheds, or population density) were found to be important drivers of microbial water quality in all primary models (Fig. 2). Specifically, the percentage of the population on septic systems was selected in 8 of the 12 primary and sensitivity analysis models, and the percentage of the population on WWTPs was selected in 4 of 12 models (Fig. 2; Table S2). WWTP variables (e.g., percent population on WWTPs and number of people on WWTPs) and number of septic tanks in watersheds consistently had positive relationships with log-transformed E. coli and B. thetaiotaomicron concentrations, in both primary and sensitivity analysis models (Fig. 2; Table S2). Despite having been selected in all models, only the baseflow sensitivity model for B. thetaiotaomicron had a statistically significant beta coefficient for one of these human pollution source variables.

### Relating seasonal changes in B. thetaiotaomicron to anticipated risks.

Temporal changes in B. thetaiotaomicron concentrations were related to anticipated infection risks using a QMRA approach to illustrate how MST, hydrologic methods, and QMRA can be collectively used. Norovirus, a human pathogen associated with fecal contamination, was used as the pathogen of interest because it is waterborne and has existing dose-response curves (22). Additional modeling details are described in Materials and Methods and in the supplemental material. Briefly, the concentrations of B. thetaiotaomicron measured in this study were related to previously reported B. thetaiotaomicron and norovirus concentrations in septic systems. The ratio of B. thetaiotaomicron to norovirus was then used to estimate norovirus concentrations in the watershed samples, assuming that norovirus was always present in the septic source (i.e., fluctuations in norovirus and B. thetaiotaomicron concentrations were not occurring and therefore the ratio was constant). Relative to mean infection risks during the baseflow season, mean infection risks for an hour of recreational activity with water ingestion in the summer rain and snowmelt seasons were decreased by 95% and 79%. A sensitivity analysis of this risk-based approach identified calculated norovirus concentration based on B. thetaiotaomicron concentrations in surface water as the most influential parameter on estimation of infection risks for all three seasons (ρ = 0.9996), followed by the pathogen concentrations in the source septic systems (ρ = 0.79) (Fig. S2).

## DISCUSSION

### Mechanistic explanations for model selection of WWTP and septic variables.

The positive relationships observed between both WWTPs and the total number of septic tanks with log-transformed E. coli and B. thetaiotaomicron indicate that contamination in the watersheds evaluated in this study may be primarily driven by human fecal sources. Identification of septic systems and WWTPs as inputs of surface water fecal contaminations in this study is consistent with existing literature (23), and there is prior evidence of WWTP and septic system contributions to the watersheds evaluated (24–27). Either a variable related to WWTPs (e.g., percentage of the population on WWTPs and number of people on WWTPs) or total number of septic systems was selected regardless of hydrologic condition, implying that inputs of human fecal contamination likely occur year-round.

**FIG 3:**
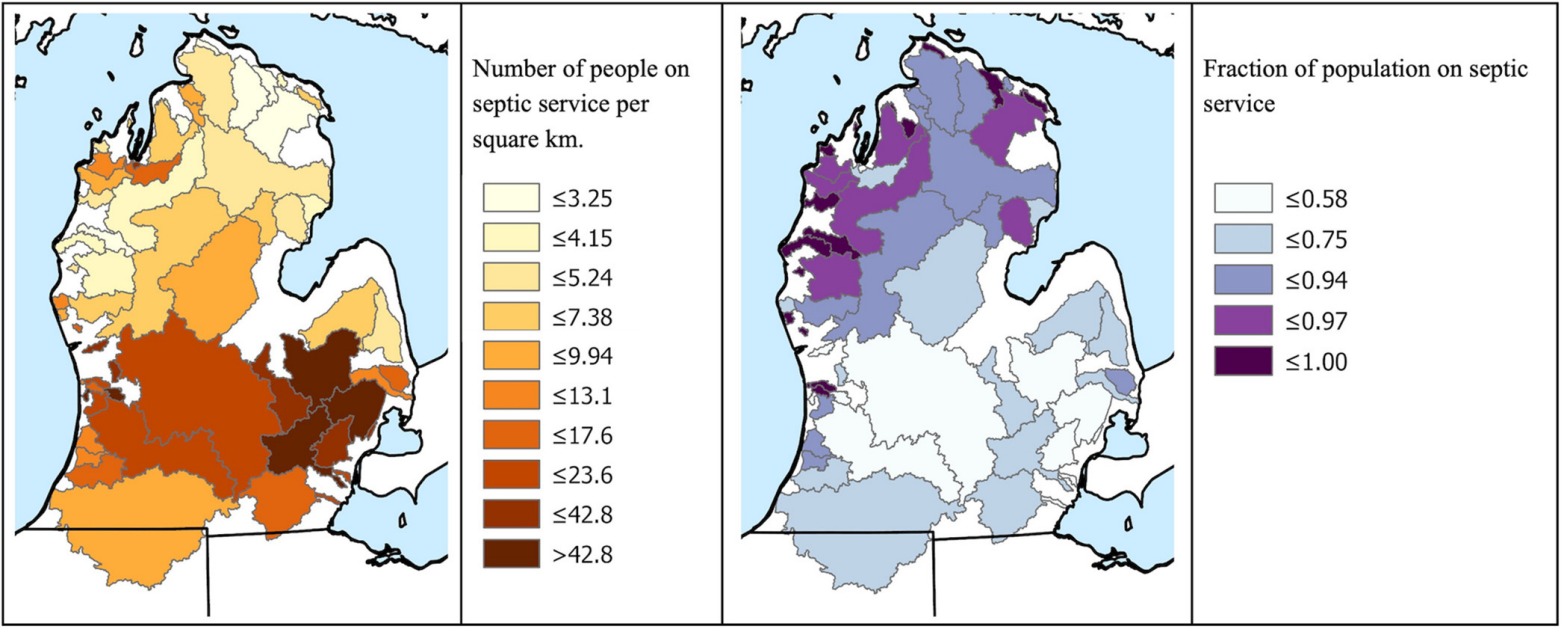
Spatial distribution of fraction of the population on septic service and number of people on septic service per square kilometer. Data are shown in quantiles, where 20% of the data are shown in each category. Each watershed sampled in this study is shown with corresponding color and gray border.

Counterintuitively, negative relationships between the percentage of the population on septic and log-transformed E. coli and B. thetaiotaomicron concentrations were observed. However, these relationships were likely confounded by structural differences in watersheds across the state, leading to covariation between underlying drivers of fecal contamination. In Michigan’s northern Lower Peninsula, most of the population that is served by septic systems (Fig. 3) lives within largely forested watersheds with limited agricultural activity. Here, a negative relationship between percentage of the population on septic and E. coli was observed that was likely driven in part by the low population density (Fig. 3). This region had relatively low E. coli concentrations overall, especially during baseflow and snowmelt seasons (Fig. 1). A similar trend was present for B. thetaiotaomicron, where concentrations were greater in watersheds in the center of the state than in the northern area of the state across seasons (Fig. 1).

In contrast, the number of septic systems in the watersheds was positively correlated with B. thetaiotaomicron concentrations during baseflow, with a significant model beta coefficient. This may be explained by B. thetaiotaomicron loading, predominately due to septic system failures. While widespread data on septic system failures are unavailable, they are likely driven by factors such as system age, usage, type, and maintenance along with soil characteristics and hydrology. Though there are spatial differences in these factors across watersheds, septic failures are a relatively rare event and should thus be viewed probabilistically. Specifically, a greater number of septic systems upstream of a sampling location means a greater probability of one or more system failures. As a result, in this study, total number of septic systems in the watershed may be a more appropriate surrogate for potential septic impact on water quality than percentage of the population on septic.

Model selection was also impacted by the methods utilized. Adaptive LASSO, like all regression shrinkage models, can underfit parameters, resulting in low predictive power (28). This statistical limitation could explain, in part, the low adjusted *R*^2^ values and relatively small beta parameter estimates in this study. However, adaptive LASSO is an appropriate approach in the case where there are many candidate variables for few outcome variables, as was the case in this study. Additionally, while collinearity among candidate explanatory variables was a challenge, ridge regression was used to shrink the weights for colinear variables (29, 30).

### Mechanisms for similarities between models of E. coli and B. thetaiotaomicron.

Some variables were shared by E. coli and B. thetaiotaomicron models for the same season, providing evidence of relationships between general fecal contamination and human-associated contamination. This also suggests that E. coli and B. thetaiotaomicron may share a source and/or follow similar fates and transport mechanisms when entering the environment. Under baseflow conditions, the percentage of the population on WWTPs was selected for both E. coli and B. thetaiotaomicron primary models (Fig. 2; Table S2). This explanatory variable had a positive relationship with E. coli and B. thetaiotaomicron concentrations, indicating the importance of direct human contributions of fecal contamination as a source for both organisms. Under snowmelt conditions, 4-day precipitation was selected in both primary models, where it had a positive relationship with E. coli and B. thetaiotaomicron concentrations (Table S2), indicating that there may be shared transport mechanisms for these organisms.

### Mechanisms for differences across seasons.

The consistent selection of a certain category of variable across seasons indicates its year-round relevance as a source or as fate and transport mechanisms. Precipitation parameters were selected across seasons in primary models (Fig. 2). However, the direction of their relationship with fecal indicators was inconsistent, implying that while it may be mechanistically important year-round, the role of precipitation in fate and transport may change. For example, in the snowmelt season, rainfall had a positive relationship with E. coli and B. thetaiotaomicron concentrations (Table S2), indicating enhanced delivery. However, in the summer rain season, rainfall had a negative relationship with log-transformed B. thetaiotaomicron concentrations (Table S2), indicating dilution effects. The positive relationship between precipitation and fecal indicators was expected, especially in conjunction with the model selection of total dissolved nitrogen: increased overland flow runoff due to precipitation in agricultural areas is expected to increase nitrogen and E. coli loading in watersheds (31). However, the negative summer relationship between precipitation and E. coli has not been reported, to our knowledge. This could be indicative of a dilution effect caused by increased overland flow rates in comparison to subsurface flow, where septic tank effluent is present. Increased surface flows may also limit the environmental persistence and regrowth of these organisms.

Elucidating the relative importance of fecal contamination sources and fate and transport mechanisms over time and across locations has the potential to reduce recreational risks (32), improve water quality surveillance efforts (33), and help prioritize fecal contamination source control (18, 34). Concentrations of the human-specific B. thetaiotaomicron were the most influential parameter related to infection risk. Thus, parameters that explain or signal seasonal changes in B. thetaiotaomicron and human fecal contamination concentrations and their relationship to pathogens would be useful for anticipating seasonal changes in risk. To better estimate absolute risks, future work could address and improve upon the assumptions within our simple QMRA model framework. For example, capturing the growth and decay dynamics of E. coli and B. thetaiotaomicron after their release from sources would allow for more accurate analysis of seasonal relationships and subsequent risk estimates. Additional data that help improve risk assessments include seasonal changes in the number of people who engage in exposure-related activities, parameters that relate differences in the transport of pathogens versus indicator organisms from sources to sampling sites, and mathematical explanations for anticipated mechanistic changes in septage inputs to surface waters as a function of season.

### Conclusions.

This study provides further evidence that WWTPs and septic systems are important, year-long sources of human fecal contamination within the evaluated watersheds. Furthermore, we show that the relationship between contamination and other explanatory variables is seasonally dependent. However, there are still uncertainties regarding the magnitude of these sources relative to other fecal contamination inputs, which is likely geographically and temporally specific. Future research is needed to quantify the impact of human-specific sources on surface water fecal contamination, accounting for geographical and temporal effects. In particular, discerning impacts from septic systems was challenging due to confounding factors and limited reporting regarding septic system integrity and usage.

The analyses from this study also demonstrate the year-long importance of precipitation in fate and transport mechanisms of fecal contamination, but inconsistencies in the direction of the relationship between precipitation and fecal indicator concentrations indicate that the role of precipitation in these mechanisms is likely seasonally driven. Other parameters, including total dissolved nitrogen, appear to be important correlates of E. coli concentrations during baseflow and snowmelt seasons and are commonly related to other watershed inputs such as agriculture. Further research is needed to understand causal mechanisms behind the relationships identified here and to quantify relationships with more statistical confidence, using larger sample sizes or other statistical methods not as limited by underfitting and low predictive power. Additionally, more data per season would provide more confidence in seasonal differences (or lack thereof) elucidated in this study.

## MATERIALS AND METHODS

### Study region and sample size.

An observational study was conducted in 64 watersheds across the state of Michigan across three hydrologic seasons: baseflow parameters were measured in fall (October 2010), snowmelt/spring thaw (March 2011), and postplanting summer rain (June 2011) (23). These intervals were selected to provide a broad range of hydrologic conditions across the contributing watersheds and within sampled streams. Details regarding sample collection timing and differences in seasonal streamflow are provided in the supplemental material.

At each site, streamflow was measured and water samples were collected as described by Verhougstraete et al. (23). Briefly, grab samples were collected from the center of each stream, at the upstream side of each access location. Biological samples were filtered and preserved within 24 h of collection. Samples for other water chemistry parameters were filtered in the field and chilled or frozen as appropriate. Laboratory analyses were performed within standard holding times and summary (23). Stream flows were either measured directly via appropriate methods or taken from 15-min data at colocated U.S. Geological Survey (USGS) streamflow gauges.

### Variables.

At each sampling location, physical and chemical parameters were measured during each sampling event. These included ammonium, calcium, chloride, dissolved oxygen, magnesium, nitrate/nitrite, total chlorophyll *a*, pheophytin-corrected chlorophyll *a*, pH, specific conductance, total dissolved nitrogen and phosphorus, sodium, potassium, dissolved organic carbon, and sulfate. Analytical methods have been described in detail by Verhougstraete et al. (23). E. coli was quantified using cultivation methods (IDEXX; Colilert), and the B. thetaiotaomicron genetic marker was measured using quantitative PCR (qPCR) (Roche; LightCycler 480 instrument) and previously published primer and probe sequences (14). Water temperature and streamflow were also measured during each sampling.

To quantify watershed precipitation prior to each sampling and static aspects of the landscape, watersheds were computed for each sampling location. For this, we used the 1/3 arc-second digital elevation model (DEM) from the National Elevation Data (NED) set, using a standard workflow in ArcMap (version 10.2): (i) the “fill” tool was run on the raw DEM, (ii) the “flowdirection” algorithm was run (which routes flow in one of the cardinal and subcardinal directions only—a D8 algorithm), (iii) the “flowaccumulation” tool was then run to make sure that sampling locations accurately fell within the DEM-generated flow network, and (iv) “watershed” was run to compute watershed areas for each sampling location.

These watersheds were then used to extract static characteristics from geographic information system (GIS) data sets that might reflect the influence of human or natural factors on B. thetaiotaomicron and E. coli concentrations. Land use and land cover data for each watershed were obtained from the USDA’s Cropland Data Layer (CDL) and the USGS’s National Land Cover Database (NLCD) 2006 values. Watershed variables associated with WWTP use, such as percentage of the population using septic tanks, number of septic systems, spatial density of septic systems, number of people on WWTPs, percentage of the population on WWTPs, and population density, were estimated using U.S. Census Bureau data as described by Luscz et al. (35).

Finally, we calculated nine spatially averaged precipitation variables within each watershed to account for precipitation totals (in millimeters) prior to sample collection in each watershed over different periods of time (6, 12, 18, and 24 h and 2, 3, 4, 6, and 8 days). Precipitation data were sourced from the North American Land Data Assimilation System (NLDAS) version 2, forcing data set A.

### Statistical methods.

Descriptive statistics were calculated for B. thetaiotaomicron and E. coli and for each sampling event: baseflow, snowmelt, and summer rain. Mixed-model analysis of variance was used to test for differences across the three seasons. Repeated measures in the same location were considered using an unstructured covariance matrix. Watersheds with missing values for candidate explanatory variables were not used in the primary or sensitivity analysis models.

The primary analysis was linear regression on log-transformed B. thetaiotaomicron and E. coli concentrations with explanatory variables selected with adaptive least absolute shrinkage and selection operator (LASSO) methods (30). Multiple statistical approaches have been used to explore environmental-microbial interactions, but to our knowledge, few have applied LASSO regression methods (36). One of the strengths of shrinkage methods includes their ability to address cases in which the number of potential explanatory variables is much larger than the number of observations (28), as is the case in this and many other stream health studies. Therefore, adaptive LASSO was used to select explanatory variables for E. coli and B. thetaiotaomicron models for all 3 seasons (6 primary models).

Ridge regression, which shrinks coefficients of correlated explanatory variables “toward each other” (29), was used to weight each candidate explanatory variable in adaptive LASSO. This is recommended for data sets in which candidate explanatory variables may be colinear, as in this case (30). Adaptive LASSO was restricted to select a number of explanatory variables that was equal to or less than the number of watersheds divided by 10 (*n*/10). For regression analysis, the outcome variables, E. coli and B. thetaiotaomicron concentrations, were log transformed. Bootstrapping methods were used to obtain 95% confidence intervals (CI) for regression parameters (beta parameter estimates) (37). Ten thousand subsets from the total data set were created using sampling with replacement, and adaptive LASSO was used to select variables from each of these subsets. If a variable was selected in more than 20% of the subset models, this variable was selected overall, and a 95% CI was reported for the beta parameters estimated per subset model.

Inclusion of zero in the 95% CI indicates uncertainty as to whether the selected variable had a relationship with the dependent variable. Therefore, exclusion of zero in the 95% CI for the beta parameter estimates indicated statistical significance at the 0.05 level. To obtain adjusted coefficients of determination (*R*^2^), linear regression was used to fit log-transformed E. coli or B. thetaiotaomicron concentrations to their respective selected variables. After explanatory variables were selected by adaptive LASSO, linear regression assumptions were evaluated (details in the supplemental material).

A sensitivity analysis was conducted for each primary model to evaluate the effect of influential watersheds on variable selection and on adjusted *R*^2^ values. Influential watersheds were identified using Cook’s D plots and were removed from the sensitivity analysis prior to performing adaptive LASSO regression. Selected variables and the adjusted *R*^2^ values for the models were then compared to those from the primary analysis that included influential watersheds. All statistical analyses were conducted using SAS 9.4 software (SAS Institute Inc., Cary, NC).

### Relative-risk estimation.

QMRA can estimate a probability of a health outcome such as infection, given a particular dose (38), and it has been used in surface water quality and recreational contexts (39–41). Differences in B. thetaiotaomicron concentrations across seasons were related to estimated relative risks of norovirus due to ingestion of water during recreational activities using a QMRA approach for relating fecal markers to reference pathogens (41). This approach has been used in multiple studies relating microbial source tracking in surface water to health outcomes (39–42). First, the B. thetaiotaomicron concentration at the watershed sampling locations ($M_{\text{sample}}$) was related to B. thetaiotaomicron concentrations at septic sources ($M_{\text{source}}$) to account for anticipated dilution from the source. A ratio of B. thetaiotaomicron concentration to norovirus concentration at the source ($P_{\text{source}}$) was then used to estimate how much norovirus would be present at the same watershed sampling location. This assumes that norovirus is always present at the source. The fraction of norovirus genome copies anticipated to be infectious ($f_{\text{infectious}}$) was then accounted for and multiplied by the estimated rate of water ingestion during recreational activities (V), informed by the work of Dorevitch et al. (43), and duration of the exposure (*d*), assumed to be equal to 1 h, consistent with another recreational water risk assessment (44), to yield an estimated dose (*D*) (equation 1):

(1) $$D=\frac{M_{\text{sample}}}{M_{\text{source}}}\times P_{\text{source}}\times f_{\text{infectious}}\times V\times d$$

While this assumes that the dilution of B. thetaiotaomicron and norovirus from septic sources would be the same, this approach was not used to calculate accurate risk. Rather, changes in risks estimated for summer rain and snowmelt seasons relative to baseflow were calculated. Data needed for a risk assessment are described in Discussion. A Monte Carlo approach was used to account for variability and uncertainty in input parameters. Explanations and sources for these distributions can be found in Table S3 and other supplemental material. As a sensitivity analysis, Spearman correlation coefficients were calculated to quantify linear relationships between input parameters and estimated infection risks. These results can be found in the supplemental material.

Distributions were fit to B. thetaiotaomicron concentrations for each season using the *fitdistrplus* package in R (version 4.0.2) (45), with details in the supplemental material. The effect of methods for handling left-censored data on distribution fitting was also explored (supplemental material). The distribution for concentrations of B. thetaiotaomicron at the source was informed by the work of Srinivasan et al. (46), and the distribution of norovirus at the source was informed by the work of Murphy et al. (47). A range of the fraction of genome copies representative of viable virus was randomly sampled from a uniform distribution with a minimum of 0.001 and a maximum of 0.01. The volume of water ingested during recreational activities was informed by the work of Dorevitch et al. (43). To account for differences in volumes ingested due to capsizing versus noncapsizing, two distributions were randomly sampled, where the volumes for capsized activities was randomly sampled 5.4% of the time to be consistent with the frequency at which capsizing occurred for these activities, according to Dorevitch et al. (43). Minimums and maximums of two uniform distributions were informed by minimum and maximum mean estimated ingested volumes (in milliliters per hour) for a variety of activities and separately for capsized versus noncapsized activity.

Dose was then related to risk of infection using an approximate beta-Poisson dose-response curve described by Van Abel et al. (22), where α=0.104 and β=32.3 (equation 2). Mean risks were used to estimate relative risks.

(2) $$P_{\text{infect}}=1-{\left(1+\frac{D}{\beta }\right)}^{-\alpha }$$
