# Supplementary material for: Detangling Seasonal Relationships of Fecal Contamination Sources and Correlates with Indicators in Michigan Watersheds
Source: Microbiol Spectr. 2022 Jun 22;10(4):e00415-22. doi: 10.1128/spectrum.00415-22 (PMC9431008; doi:10.1128/spectrum.00415-22)
Supplement: Supplemental file 1 — Supplemental material. Download spectrum.00415-22-s0001.pdf, PDF file, 0.4 MB [file spectrum.00415-22-s0001.pdf]

Supplemental Materials for

Detangling seasonal relationships of fecal contamination sources and correlates with indicators in Michigan watersheds

Amanda M. Wilson[1], Sherry L. Martin[2], Marc P. Verhougstraete[1*], Anthony D. Kendall[2], Amy G. Zimmer-Faust[3], Joan B. Rose[4], Melanie L. Bell[5], David W. Hyndman[2]

1. Department of Community, Environment & Policy, Mel and Enid Zuckerman College of Public Health, University of Arizona, Tucson, Arizona
2. Department of Earth and Environmental Sciences, College of Natural Science, Michigan State University, East Lansing, Michigan
3. Southern California Coastal Water Research Project Authority, Costa Mesa, CA
4. Department of Fisheries and Wildlife, College of Agriculture and Natural Resources, Michigan State University, East Lansing, Michigan
5. Department of Epidemiology and Biostatistics, Mel and Enid Zuckerman College of Public Health, University of Arizona, Tucson, Arizona

Contents

Stream Characteristics by Season and Sampling Design 3

Site Selection 4

Evaluation of Assumptions for Linear Regression 4

Observations and Missing Data 4

Distribution Fitting 5

Table S1 6

Table S2. 8

Table S3. 14

Figure S1 15

Figure S2 16

Stream Characteristics by Season and Sampling Design

During the baseflow period, groundwater discharge to surface waters is the predominant source of streamflow, thus samples taken during this interval reflect groundwater sources to a greater degree than surface sources. Following a typical seasonal snowpack accumulation, the snowmelt/spring thaw sampling period captured high streamflows resulting both from relatively high groundwater discharge and surface runoff over saturated and in some cases frozen ground. The summer rain period was selected in order to capture high streamflows resulting from intense late spring/early summer rain events following planting and fertilization of agricultural fields.

The sampling design was a hybrid synoptic/event driven scheme, wherein the lower peninsula of the State of Michigan was subdivided into major quadrants, and each quadrant was sampled over a period of one or two days while certain hydrologic conditions were met. The baseflow samples were collected between October 1 and 4, 2010, with a secondary collection on October 12 and 13. All samples were collected following several days of little to no rainfall, during a predominantly dry time of year. The snowmelt sampling was conducted March 4-7, 2011 in the southern quadrants of the State and March 19-23 in the northern quadrants. Dates were selected to capture significant warming periods during which snowpack melt was observed. The summer rainfall event required that crops had been planted, with sufficient time to allow for post-planting “side dress” fertilization—occurring typically a few weeks after planting. Additionally, a large enough rainfall event, of more than 1 inch, was required in order to drive streamflows higher and assure that some direct overland flow to streams would have occurred. This resulted in a first sampling event from June 1-4 2011, followed by a second from June 24 to 28. Details regarding site selection can be found in the supplemental materials.

Site Selection

Site selection was split into two primary objectives: 1) capture the bulk of streamflow from Michigan's Lower Peninsula by sampling the largest stream systems, and 2) capture the variability in land use and landscape characteristics present across Michigan's tributary water via a stratified random sampling of smaller order watersheds. The end result was 64 sites, split roughly evenly between both large and small watersheds. Within each watershed, the most-downstream (upstream from the Great Lakes) easily accessible points were identified. This was subject to the limitation that streams were flowing without impedance from the downstream receiving Great Lakes waters. Complete sampling location details are provided by Verhougstraete et al. (2015).

Evaluation of Assumptions for Linear Regression

Linear relationships between each explanatory variable and log-transformed microbial indicator concentrations were investigated by examining locally estimated scatterplot smoothing (loess) plots. Independence was assumed for all observations, since measures collected at different times for the same watershed were not used together in any model. Homoscedasticity was checked by investigating scatter plots of residuals versus explanatory variable values. Q-Q plots were used to check for normality in the residuals.

Observations and Missing Data

For baseflow and snowmelt, there were missing data (baseflow $\leq$ 5 observations, snowmelt: $\leq$ 12 observations) for pH, specific conductance, non-purgeable organic carbon, total dissolved nitrogen, nitrate, alkalinity, chlorine, sulfate, calcium, magnesium, sodium, potassium, ammonium, and total chlorophyll *a*, and phaeophytin corrected chlorophyll *a*. For summer rain, there were missing data (1 observation) for total phosphorus, total dissolved phosphorus, total

phosphorous to total nitrogen ratio, ammonium, and soluble reactive phosphorus in addition to variables for which there were missing data in baseflow and snowmelt data sets. The total number of observations (i.e. watersheds) used in primary analysis were 56, 46, and 60 for the baseflow, snowmelt, and post-planting rain models, respectively.

Distribution Fitting

Distributions were fit to *B. theta* and *E. coli* concentrations by season. Candidate distributions included lognormal and weibull. For left-censored values, which only occurred for snowmelt and summer rain seasons, the limit of detection/2 (400 gc/100mL) was used in place of censored values. The effect of this on distribution was fitting by comparing distribution parameters using the *fitdistcens* and *fitdist* functions in the 'fitdistrplus' R package, and there were negligible differences. This is likely due, in part, to low to medium amounts of left-censoring (snowmelt: 6.25%, summer rain: 36.5%).

Distribution fits between candidate distributions were compared visually and using a Kolmogorov-Smirnov test. For baseflow, while both fit lognormal and weibull distributions were not statistically significantly different from the data, the lognormal distribution visually appeared to be a better fit (Figure S1). This was also true for the snowmelt data. For summer rain, both the weibull and lognormal distributions were statistically significantly different from the data. However, the weibull distribution visually appeared to be a better fit (Figure S1).

Table S1. Descriptive statistics, median (interquartile range), and Mixed Model ANOVA P values of continuous variables of the full watersheds with statistically significant differences across three seasons (baseflow, snowmelt, and summer rain) and *Escherichia coli* concentrations*

Variable	Season			P-value
	Baseflow	Snowmelt	Summer Rain	
E. coli concentration (MPN/100 mL)	6.51×10^{1} (1.38×10^{2})	3.36×10^{1} (1.49×10^{2})	8.42×10^{1} (1.53×10^{2})	0.0018**
B. theta cell equivalent (ce)/100 mL	1.47×10^{5} (2.83×10^{5})	8.07×10^{3} (7.76 x 103)	1.37×10^{3} (2.14×10^{3})	<0.001**
Temperature (Celsius)	1.38×10^{1} (3.71×10^{0})	2.24×10^{0} (2.08×10^{0})	1.89×10^{1} (2.80×10^{0})	<0.0001
Dissolved oxygen (mg/L)	9.54×10^{0} (2.37×10^{0})	9.81×10^{0} (3.34×10^{0})	8.00×10^{0} (1.66×10^{0})	<0.0001
6-hour precipitation (mm)	0.00×10^{0} (0.00×10^{0})	6.93×10^{-2} (1.06×10^{0})	0.00×10^{0} (0.00×10^{0})	<0.0001
12-hour precipitation (mm)	0.00×10^{0} (0.00×10^{0})	1.47×10^{-1} (2.18×10^{0})	0.00×10^{0} (0.00×10^{0})	<0.0001
24-hour precipitation (mm)	0.00×10^{0} (2.7×10^{0})	2.05×10^{0} (6.67×10^{0})	0.00×10^{0} (2.84×10^{-2})	<0.0001
2-day precipitation (mm)	1.09×10^{0} (5.57×10^{0})	8.92×10^{0} (1.66×10^{1})	0.00×10^{0} (1.20×10^{0})	<0.0001
3-day precipitation (mm)	2.15×10^{0} (6.66×10^{0})	1.18×10^{1} (1.84×10^{1})	1.14×10^{0} (5.13×10^{0})	<0.0001
4-day precipitation(mm)	2.40×10^{0} (5.99×10^{0})	1.20×10^{1} (1.83×10^{1})	3.72×10^{0} (2.06×10^{1})	<0.0001
6-day precipitation(mm)	3.40×10^{0} (5.46×10^{0})	1.72×10^{1} (1.90×10^{1})	2.98×10^{1} (3.80×10^{1})	<0.0001
8-day precipitation (mm)	4.24×10^{0} (6.33×10^{0})	1.81×10^{1} (1.88×10^{1})	4.99×10^{1} (2.95×10^{1})	<0.0001
Flow rate (m^3/sec)	1.57×10^{0} (6.63×10^{0})	5.64×10^{0} (2.36×10^{1})	4.72×10^{0} (3.25×10^{1})	0.0003

Total phosphorus (µg/L)	2.55×10^{1} (2.69×10^{1})	7.65×10^{1} (6.84×10^{1})	3.57×10^{1} (4.25×10^{1})	<0.0001
Total dissolved phosphorus (µg/L)	1.59×10^{1} (1.59×10^{1})	3.77×10^{1} (3.36×10^{1})	2.2×10^{1} (2.4×10^{1})	0.0004
Ammonia (µg/L)	5.12×10^{0} (2.95×10^{1})	6.16×10^{1} (9.62×10^{1})	1.80×10^{1} (2.20×10^{1})	<0.0001
Soluble reactive phosphorus (µg/L)	9.57×10^{0} (1.19×10^{1})	9.27×10^{0} (2.70×10^{1})	6.19×10^{0} (1.02×10^{1})	0.0069
pH	8.21×10^{0} (1.30×10^{-1})	7.97×10^{0} (2.19×10^{-1})	8.11×10^{0} (2.10×10^{-1})	<0.0001
Chlorine (µg/L)	2.09×10^{1} (3.79×10^{1})	1.90×10^{1} (2.69×10^{1})	1.44×10^{1} (2.51×10^{1})	0.0053
Non-purgeable organic carbon (µg/L)	5.01×10^{0} (3.09×10^{0})	6.81×10^{0} (2.84×10^{0})	1.67×10^{1} (1.96×10^{1})	<0.0001
Total dissolved nitrogen (µg/L)	6.58×10^{-1} (7.69×10^{-1})	1.22×10^{0} (2.04×10^{0})	1.13×10^{0} (1.21×10^{0})	0.0026
Nitrate (µg/L)	2.58×10^{-1} (7.79×10^{-1})	9.09×10^{-1} (1.96×10^{0})	5.51×10^{-1} (1.30×10^{0})	0.0035
Alkalinity (mEq/L)	3.50×10^{3} (1.28×10^{3})	3.03×10^{3} (1.09×10^{3})	3.29×10^{3} (1.09×10^{3})	<0.0001
Magnesium (µg/L)	1.68×10^{1} (1.07×10^{1})	1.37×10^{1} (6.29×10^{0})	1.40×10^{1} (5.48×10^{0})	0.00077
Total chlorophyll *a* (µg/L)	6.18×10^{-1} (1.81×10^{0})	5.86×10^{-1} (7.59×10^{-1})	7.24×10^{-1} (6.00×10^{-1})	0.0099
Phaeophytin corrected chlorophyll *a* (µg/L)	3.01×10^{-1} (9.27×10^{-1})	3.51×10^{-1} (4.33×10^{-1})	3.19×10^{-1} (2.93×10^{-1})	0.012

* Missing values were excluded from summary statistic calculations. The mixed model comparing 18-hr precipitation did not converge.
** P-values for *E. coli* and *B. theta* reflect differences in log concentration

Table S2. Summary of spring, summer, and fall sensitivity analysis for adaptive lasso coefficient estimates of selected variables in both primary and sensitivity analysis with respective R^2 and adjusted R^2, with and without influential watersheds, where bolded parameters indicate confidence intervals that do not include zero

E. coli							
**Season**	Primary Model			Sensitivity Analysis Model			
	Selected Variables	Estimated Coefficient (95% CI)	Adjusted R^2 (R^2)	Selected Variables	Estimated Coefficient (95% CI)	Number of Influential Watersheds	Adjusted R^2 (R^2)
**Baseflow**	**Total dissolved nitrogen**	**3.1×10^{-1} (1.1×10^{-2}, 5.3×10^{-1})**	0.23 (0.28)	6-day precipitation	1.7×10^{-2} (0.0×10^{0}, 5.6×10^{-2})	2	0.12 (0.16)
	Calcium	2.7×10^{-3} (0.0×10^{0}, 1.3×10^{-2})		**Sodium**	**1.4×10^{-2} (7.4×10^{-3}, 2.6×10^{-2})**		
	Percent population on septic	-1.5×10^{0} (-3.4×10^{0}, 0.0×10^{0})		Percent population on septic	-3.9×10^{-1} (-1.7×10^{0}, 7.1×10^{-1})		
	Percent population on WWTP	8.7×10^{-1}					

		(0.0 x 10^{0}, 3.2 x 10^{0})					
**Snowmelt**	4-day precipitation	1.3 x 10^{-2} (0.0 x 10^{0}, 5.1 x 10^{-2})	0.43 (0.46)	**4-day precipitation**	**4.7 x 10^{-2} (4.4 x 10^{-3}, 8.2 x 10^{-2})**	3	0.51 (0.54)
	**Total dissolved nitrogen**	**1.8 x 10^{-1} (1.8 x 10^{-2}, 4.6 x 10^{-1})**		Total dissolved nitrogen	2.0 x 10^{-1} (0.0 x 10^{0}, 3.8 x 10^{-1})		
	Percent population on septic	-2.9 x 10^{0} (-5.2 x 10^{0}, 0.0 x 10^{0})		Percent population on septic	-2.4 x 10^{0} (-4.4 x 10^{0}, 0.0 x 10^{0})		
**Summer rain**	Calcium	1.1 x 10^{-2} (0.0 x 10^{0}, 2.6 x 10^{-2})	0.28 (0.35)	**Total dissolved nitrogen**	**2.2 x 10^{-1} (1.0 x 10^{-1}, 3.5 x 10^{-1})**	4	0.36 (0.40)
	Sodium	9.4 x 10^{-3} (0.0 x 10^{0}, 1.7 x 10^{-2})		**Chloride**	**1.4 x 10^{-2} (5.3 x 10^{-3}, 2.0 x 10^{-2})**		
	Percent population on septic	-6.9 x 10^{-1}		Calcium	1.1 x 10^{-3}		

		(-1.8×10^{0}, 0.0×10^{0})			(0.0×10^{0}, 8.8×10^{-e})		
	Winter Wheat CDL Value 24	4.6×10^{0} (0.0×10^{0}, 1.3×10^{1})		Number of People on WWTP	6.2×10^{-5} (0.0×10^{0}, 5.4×10^{-4})		
	Other Crops CDL Value 44	5.0×10^{0} (0.0×10^{0}, 3.2×10^{1})					
	Mean percent of impervious surfaces	5.1×10^{-3} (0.0×10^{0}, 3.5×10^{-2})					
B. theta							
**Season**	Primary Model			Sensitivity Analysis Model			
	Selected Variables	Estimated Coefficient (95% CI)	Adjusted R^2 (R^2)	Selected Variables	Estimated Coefficient (95% CI)	Number of Influential Watersheds	Adjusted R^2 (R^2)
**Baseflow**	Phaeophytin corrected chlorophyll *a*	1.0×10^{-1}	0.22 (0.29)	4-day precipitation	6.7×10^{-3} (0.0×10^{0}, 3.7×10^{-2})	5	0.16 (0.22)

		(0.0 x 10^{0}, 2.5 x 10^{-1})					
	Chloride	1.0 x 10^{-3} (0.0 x 10^{0}, 5.3 x 10^{-3})		Sulfate	1.9 x 10^{-3} (0.0 x 10^{0}, 6.6 x 10^{-3})		
	Magnesium	1.3 x 10^{-2} (0.0 x 10^{0}, 4.5 x 10^{-2})		**Number of septic systems in the watershed**	**7.3 x 10^{-6} (3.8 x 10^{-6}, 1.4 x 10^{-5})**		
	Population density	-5.7 x 10^{-4} (-1.4 x 10^{-3}, 0.0 x 10^{0})		Population density	-6.5 x 10^{-5} (-5.5 x $10^{-4,}$ 0.0 x 10^{0})		
	Percent of the population on WWTP	8.1 x 10^{-1} (0.0 x 10^{0}, 2.1 x 10^{0})					
**Snowmelt**	4-day precipitation	3.1 x 10^{-3} (0.0 x 10^{0}, 1.6 x 10^{-2})	0.19 (0.24)	Percent of the population on WWTP	1.4 x 10^{0} (0.0 x 10^{0}, 2.7 x 10^{0})	4	0.13 (0.16)

	Flow rate	4.4×10^{-4} (0.0×10^{0}, 3.3×10^{-3})		Percent population on septic	-4.6×10^{-1} (-2.6×10^{0}, 0.0×10^{0})		
	Total number of people using septic tanks	3.7×10^{-4} (0.0×10^{0}, 1.2×10^{-3})					
	Percent population on WWTP	2.1×10^{0} (0.0×10^{0}, 3.3×10^{0})					
**Summer rain**	Phaeophytin corrected chlorophyll *a*	9.3×10^{-2} (0.0×10^{0}, 3.7×10^{-1})	0.17 (0.24)	Total chlorophyll *a*	9.8×10^{-2} (0.0×10^{0}, 2.2×10^{-1})	2	0.14 (0.22)
	24-hr precipitation	-3.0×10^{-2} (-1.1×10^{-1}, 0.0×10^{0})		**Total dissolved nitrogen**	**-1.6×10^{-1} (-2.5×10^{-1}, -5.1×10^{-2})**		
	Saturated hydraulic conductivity in the watershed	-8.0×10^{-1} (-2.0×10^{0}, 0.0×10^{0})		Population density	7.8×10^{-5} (0.0×10^{0}, 4.3×10^{-4})		

	Percent population on septic	-8.2×10^{-1} (-1.5×10^{0}, 0.0×10^{0})		Percent population on septic	-4.4×10^{-1} (-1.3×10^{0}, 0.0×10^{0})		
	Winter Wheat CDL Value 24	-1.6×10^{0} (-8.5×10^{0}, 0.0×10^{0})		Total number of people on WWTP	3.8×10^{-5} (0.0×10^{0}, 3.2×10^{-4})		

Table S3. Distributions and sources for relative risk estimation and Spearman correlation coefficients for input parameter relationships with infection risk

Variable	Description	Distribution or Point Value	Distribution or Point Value Source
M_{source}	*B. theta* concentrations at the source, ($\log_{10}$ gc/100mL)	Normal (mean=7.26, sd=0.24), left-truncated at 0	[1]
M_{sample}	*B. theta* concentrations at sampling locations, (gc/100mL)	Baseflow<br>Lognormal<br>(meanlog=11.85, sdlog=1.03)<br><br>Snowmelt<br>Lognormal<br>(meanlog=8.85, sdlog=1.10)<br><br>Summer rain<br>Weibull<br>(shape=1.18, scale=1884.9)	This study
P_{source}	Norovirus concentrations at the source, (gl/L)	Weibull (shape=0.6267, scale=23.72)	[2]
$f_{infectious}$	Fraction of norovirus gc assumed to be infectious, unitless	Uniform (min=5×10^{-4}, max=1×10^{-2})	[3]
$V*$	Water ingestion rate, mL/hr	Non-capsized activity (94.6% of iterations)<br>Uniform (min=3.6, 3.9)<br><br>Capsized activity (5.4% of iterations)<br>Uniform (3.5, 6)	[4]
d	Duration of	1 hour	Assumed; [5]

	recreational activity, min		
β	Dose-response curve parameters	0.104	[6]
α		32.3	

*To capture ingestion volumes for capsized and noncapsized activities, the distribution of capsized ingestion volumes was randomly sampled for 5.4% of iterations, and the distribution for noncapsized ingestion volumes was randomly sampled for 94.6% of iterations. Activities for ingested volumes included canoeing, kayaking, rowing, and fishing.

Baseflow	Snowmelt	Summer Rain

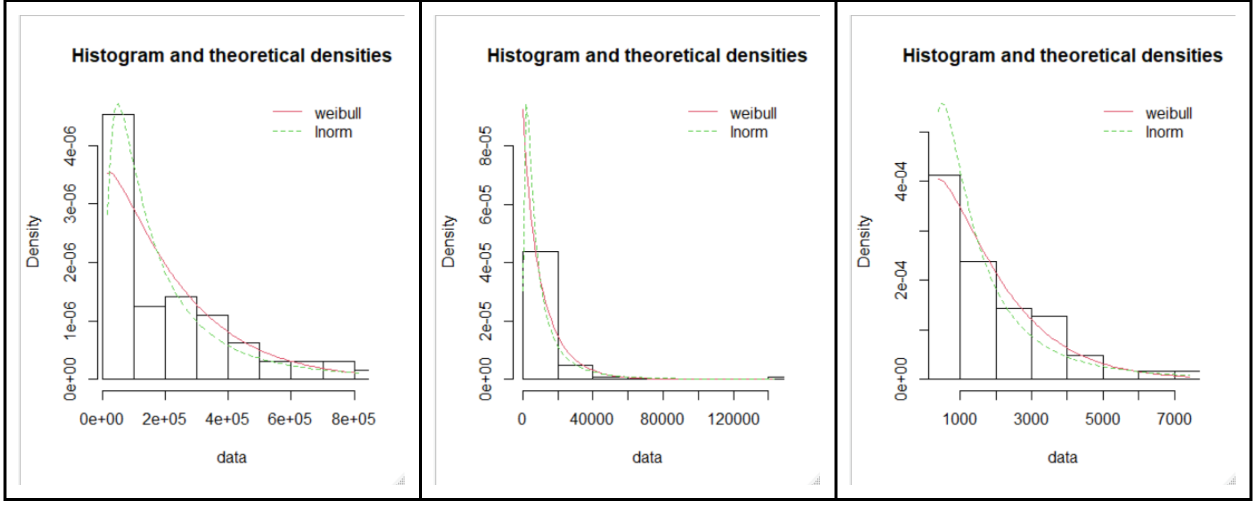

Figure S1. Comparisons of distribution fits to *B. theta* concentrations per season

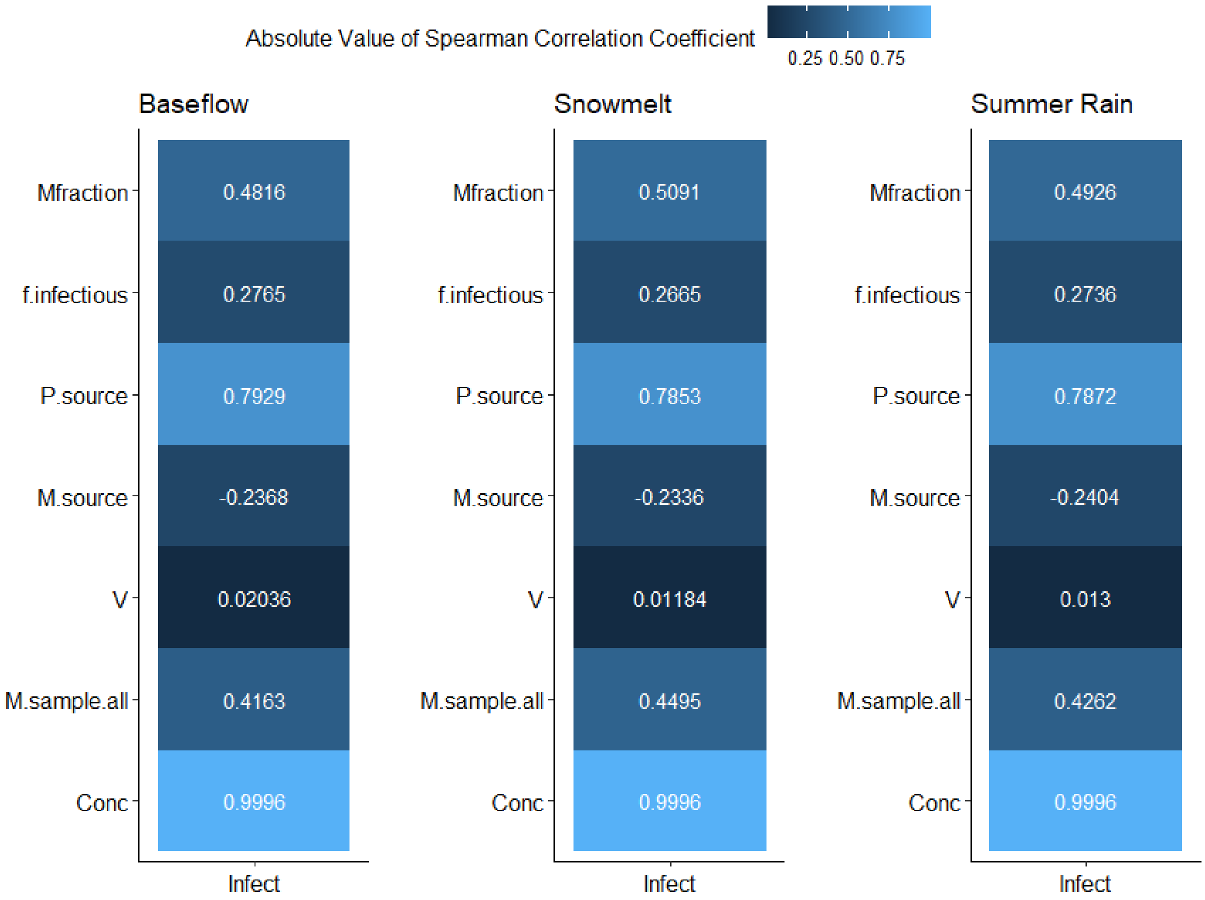

Figure S2. Spearman correlation coefficients for relative risk estimates*

*P.source = concentration of norovirus at the source (gc/100mL), M. source = concentration of *B. theta* at the source (gc/100mL), V = volume of water ingested during the activity, M.sample.all= concentration of *B. theta* at the sampling location, Mfraction = M.sample.all/M.source, Conc=concentration of norovirus at the sampling location, f.infectious=fraction of genome copies that represent infectious virus, Infect=infection risk

References

1. Srinivasan S, Aslan A, Xagoraraki I, Alocilja E, Rose JB. Escherichia coli, enterococci, and Bacteroides thetaiotaomicron qPCR signals through wastewater and septage treatment. *Water Research*. 2011;45(8):2561-2572. doi:10.1016/j.watres.2011.02.010

2. Murphy HM, Thomas MK, Schmidt PJ, Medeiros DT, McFadyen S, Pintar KDM. Estimating the burden of acute gastrointestinal illness due to Giardia, Cryptosporidium, Campylobacter, E. Coli O157 and norovirus associated with private wells and small water systems in Canada. *Epidemiology and Infection*. 2016;144(7):1355-1370. doi:10.1017/S0950268815002071

3. Sinclair RG, Choi CY, Riley MR, Gerba CP. Pathogen Surveillance Through Monitoring of Sewer Systems. In: *Advances in Applied Microbiology*. Elsevier Inc.; 2008. doi:10.1016/S0065-2164(08)00609-6

4. Dorevitch S, Panthi S, Huang Y, et al. Water ingestion during water recreation. *Water Research*. 2011;45(5):2020-2028. doi:10.1016/j.watres.2010.12.006

5. Gitter A, Mena KD, Wagner KL, et al. Human health risks associated with recreational waters: Preliminary approach of integrating quantitative microbial risk assessment with microbial source tracking. *Water (Switzerland)*. 2020;12(2):1-16. doi:10.3390/w12020327

6. Van Abel N, Schoen ME, Kissel JC, Meschke JS. Comparison of risk predicted by multiple norovirus dose-response models and implications for quantitative microbial risk assessment. *Risk Analysis*. 2017;37(2):245-264. doi:10.1111/risa.12616
